# Identification of functional genetic variation in exome sequence analysis

**DOI:** 10.1186/1753-6561-5-S9-S13

**Published:** 2011-11-29

**Authors:** Andrew Jaffe, Genevieve Wojcik, Audrey Chu, Asieh Golozar, Ankit Maroo, Priya Duggal, Alison P Klein

**Affiliations:** 1 Department of Epidemiology, Johns Hopkins Bloomberg School of Public Health, 615 N Wolfe Street, Baltimore, MD 21205, USA; 2 Division of Cancer Epidemiology and Genetics, National Cancer Institute, National Institutes of Health, 6120 Executive Boulevard, Bethesda, MD 20892, USA; 3 Department of Oncology, Sidney Kimmel Comprehensive Cancer Center, Johns Hopkins School of Medicine, Harry and Jeanette Weinberg Building, 401 North Broadway, Baltimore, MD 21231, USA; 4 Department of Pathology, Johns Hopkins School of Medicine, 600 North Wolfe Street, Baltimore, MD 21287, USA

## Abstract

Recent technological advances have allowed us to study individual genomes at a base-pair resolution and have demonstrated that the average exome harbors more than 15,000 genetic variants. However, our ability to understand the biological significance of the identified variants and to connect these observed variants with phenotypes is limited. The first step in this process is to identify genetic variation that is likely to result in changes to protein structure and function, because detailed studies, either population based or functional, for each of the identified variants are not practicable. Therefore algorithms that yield valid predictions of a variant’s functional significance are needed. Over the past decade, several programs have been developed to predict the probability that an observed sequence variant will have a deleterious effect on protein function. These algorithms range from empirical programs that classify using known biochemical properties to statistical algorithms trained using a variety of data sources, including sequence conservation data, biochemical properties, and functional data. Using data from the pilot3 study of the 1000 Genomes Project available through Genetic Analysis Workshop 17, we compared the results of four programs (SIFT, PolyPhen, MAPP, and VarioWatch) used to predict the functional relevance of variants in 101 genes. Analysis was conducted without knowledge of the simulation model. Agreement between programs was modest ranging from 59.4% to 71.4% and only 3.5% of variants were classified as deleterious and 10.9% as tolerated across all four programs.

## Background

Identification of genetic variation that affects human health has resulted in improvements in public health through the development of better treatments, diagnostics, and preventive strategies. One major source of this genetic variation is single-base changes in the DNA sequence, some of which lead to alterations in protein structure and function. These single-nucleotide polymorphisms (SNPs) occur in the genome approximately once every 1,200–1,500 base pairs and are nonrandomly distributed [1]. Variants that occur in the protein coding regions are further classified into synonymous and nonsynonymous variants. Synonymous SNPs are defined as DNA changes without an associated change in the amino acid sequence, and nonsynonymous SNPs are defined as DNA changes that result in an amino acid substitution or insertion of a stop codon. In one of the first examples of complete sequence analysis of an individual genome, 10,569 nonsynonymous SNPs were found, of which only 8,996 had been previously observed [2]. This discovery of 1,573 novel nonsynonymous SNPs demonstrates the magnitude of novel information that can be gained from genome sequencing.

Although it may be difficult to know with certainty the effect of a single amino acid change on protein function, some understanding can be gained through our knowledge of protein biochemistry or gene sequence itself. One sequence-based feature that can be examined is whether or not the sequence variant results in a codon change. If a nonsynonymous SNP results in a stop codon, then the variant is likely to be deleterious, unless this premature stop occurs near the end of a gene. The effect of an amino acid substitution can range from negligible to severe depending on the biochemical properties of the substituted amino acid. In addition to examining the amino acids found at the mutation site, one can use phylogeny to determine conserved sequences within a gene. Conserved sites are likely to have arisen because genetic variation in these regions is not tolerated across species as a result of strong negative selective pressure against variation in these regions. Thus variants that occur in nonconserved regions are more likely to be tolerated.

In the past decade several programs have been developed with the goal of predicting whether or not an observed sequence variant is likely to have a deleterious effect on the protein product. These programs combine data from a variety of sources and use differing computational algorithms to estimate the probability that an observed genetic variant is deleterious. In this paper, we compare the findings of four nucleotide- and amino acid–based algorithms aimed at predicting the effect of an observed nonsynonymous sequence variation. We predict functional results for nonsynonymous SNPs using the nucleotide version of SIFT, PolyPhen-2, VarioWatch, and MAPP. Based on the predictions made by the various programs, we classify variants as deleterious or tolerated and calculate the agreement between programs.

## Methods

The data set consists of variants observed in the individual sequences as part of phase 3 of the 1000 Genomes Project, as released for Genetic Analysis Workshop 17 (GAW17). We selected 101 autosomal genes and analyzed only those nonsynonymous SNPs that caused a missense mutation in the subsequent amino acid. Overall, 3,781 nonsynonymous SNPs were identified within these genes. We identified amino acid changes using the ANNOVAR program [3] by mapping the genomic coordinates of each variant to the RefSeq gene database (build hg18/36.3). The result was an average of 37.4 nonsynonymous variants (standard deviation: 23.4 variants) per gene. There were 1,867 (49.4%) private variants (one copy present, minor allele frequency [MAF] < 0.001), 1,310 (34.6%) very rare variants (0.001 < MAF < 0.01), 371(9.8%) rare variants (0.01 < MAF < 0.05), and 233 (6.2%) common variants (MAF > 0.05).

The functional analysis programs we examined (SIFT, PolyPhen-2, MAPP, and VarioWatch) fall into two categories: nucleotide-based and amino acid–based. Some programs provide probabilities, whereas others classify their predictions into levels of functional threats. To carry out this analysis, we dichotomize predictions as either tolerated or deleterious. We also give an overview of each program.

To compare our results to a gold standard, we examined the entire GAW17 data set to identify variants that had been previously analyzed in experimental models providing true functional results. Because the simulated GAW17 data do not represent true biological function, the simulated data could not be used as a gold standard. We identified functional variants by searching for information by gene name for each amino acid change in the Online Mendelian Inheritance in Man (OMIM) database [4] along with the term “functional” using an R program. We were able to identify 19 functional variants using this method, 13 of which were verified as tested in mouse or cell line models. We identified an additional two variants (in *BRCA2* and *MFTRR*) by searching the literature.

### Programs

By querying data from five databases (Ensembl, UCSC BLAT, Rescue-ESE, Fas-ESS, and SIFT), VarioWatch provides functional annotation for SNPs. We used the following criteria, as implemented in the program: (1) whether or not the mutation affects the protein structure, (2) whether the SNP is in an exon-splicing enhancer or silencer, and (3) whether or not the SNPs abolish the protein domain [5]. Once VarioWatch is provided with information on variant location, it returns predictions in terms of high, medium, and low levels of threat using the three criteria. In our analyses we classified VarioWatch’s very high and high-level threats as deleterious and its low- and medium-level threats as tolerated.

Multivariate Analysis of Protein Polymorphism (MAPP) is a phylogeny-based Java-script program that estimates the average deviation from six physiochemical properties (hydropathy, polarity, charge, volume, free energy in alpha-helix conformation, and free energy in beta-strand conformation) at an amino acid position across species to assess the effect of a substitution at a particular amino acid site [6]. Provided with a set of pre-aligned orthologous protein sequences and a tree relating the sequences, MAPP can be used to estimate the effect of a newly detected polymorphism on protein function. We obtained homologous sequences for the same 10 species from the UCSC database for each gene (rhesus, mouse, dog, elephant, opossum, platypus, chicken, stickleback, lizard, *Xenopus tropicalis*). For this analysis, the severe and moderate levels were classified as deleterious, and the minor level was classified as tolerated.

The Sorting Intolerant from Tolerant (SIFT) program is a tool based on sequence homology and physiochemical properties that detects variation from the alignment and gives a probability score of the mutation being deleterious as a measure of a substitution affecting the protein function [7,8]. A semiautomated approach allows the user to input homologous sequences or alignments to improve accuracy. Input includes the reference and variant nucleotide and their chromosomal position. SIFT provides binary classification of variants as either tolerated or deleterious.

The PolyPhen-2 program predicts the effect of a variant using eight sequence-based and three structure-based features previously selected by an iterative greedy algorithm [9,10]. Sequence-based features include whether the variant is in an active or binding site and whether other functionally important regions or motifs are present. Sequences are pulled from Uniprot. Structural components include whether or not the variant alters the polarity of the structure, potentially changing the hydrophobic core of a protein, and whether its interactions are with itself or with other proteins. The functional prediction is determined by a naïve Bayes classifier into deleterious and nondeleterious groups. Input is amino acid changes with the reference and variant amino acid and their position in the protein sequence. The output levels of possibly damaging and probably damaging were classified as deleterious for this analysis, with the benign level being classified as tolerated.

## Results

We obtained moderate levels of agreement for the four programs, with agreement ranging from 59% to 71% (Table 1). The highest agreement was observed between MAPP and PolyPhen-2: 71%. The lowest agreement, 59%, was observed between VarioWatch and MAPP.

**Table 1:** Agreement between prediction programs

Program	MAPP (%)	SIFT (%)	VarioWatch (%)	PolyPhen-2 (%)
MAPP	100			
SIFT	64.6	100		
VarioWatch	59.4	62.9	100	
PolyPhen-2	71.4	64.2	62.9	100

For the off-diagonal values, this table looks at pairwise agreements between the four prediction programs in predicting whether a variant is tolerated or deleterious. For example, 64.6% of the time, MAPP and SIFT agreed on their prediction, whereas VarioWatch and SIFT only agreed 62.9% of the time. The highest agreement is between MAPP and PolyPhen-2, and the lowest agreement is between MAPP and VarioWatch.

We also saw trends in the leniency of the predictions across the 101 genes, with some programs categorizing fewer variants as deleterious compared to others. To further examine the correlation between programs, we calculated the proportion of variants classified as deleterious per program. In addition, we examined the conditional probability that a variant classified as deleterious by a given program would also be classified as deleterious in a second program. For example, in Table 2, MAPP classified 1,472 of the 3,199 variants for which predictions were available as deleterious. Of these 1,472, 65.4% were also classified as deleterious by VarioWatch [i.e., Prob(deleterious in VarioWatch | deleterious in MAPP)]. Conversely, of the 1,882 variants that were classified as deleterious in VarioWatch (*n* = 1,882), only 54.6% were also classified as deleterious by MAPP. Overall, 135 (3.57%) variants were called deleterious by all programs, whereas only 414 (10.9%) were classified as tolerant by all four programs. Of the 3,781 variants examined, 876 were classified as deleterious by three or more programs, and 1,274 were classified as tolerated by three or four programs. This suggests that these programs are more likely to agree on benign variants than on potentially damaging variants.

**Table 2:** Comparison of deleterious SNPs across programs

Program	Number of variants classified	Deleterious		Conditional probability of pairwise prediction			
		
		Number	%	MAPP (%)	SIFT (%)	VarioWatch (%)	PolyPhen-2 (%)
MAPP	3,199	1,472	46.0	100	58	65	78

SIFT	3,562	1,603	45.0	62	100	71	69
VarioWatch	3,429	1,882	54.9	55	58	100	64
PolyPhen-2	3,333	1,757	52.7	65	58	67	100

Each program characterizes a different number of deleterious alleles. The pairwise prediction is calculated by conditioning on the probability of a program predicting a variant as deleterious, given that the comparison program already has predicted it as deleterious. For example, in the first row, among those 1,472 variants that MAPP classifies as deleterious, only 58% are also classified as deleterious by SIFT.

We next compared the predictions obtained from these four programs to the results of the functional studies conducted on the 15 genetic variants for which functional data were available. Of these 15 variants, 13 had been demonstrated to result in a loss of function. However, only 6 of these 13 loss-of-function mutations were predicted to have a deleterious effect on protein function by all four programs (Table 3). Interestingly, even within these known functional variants, no variant was predicted as benign or tolerated by all four programs. Of the four programs, PolyPhen-2 had the greatest accuracy, predicting the function of 11 of the 15 variants correctly. VarioWatch and SIFT predicted 10 out of 15 correctly, and MAPP predicted 9 out of 15 correctly.

**Table 3:** Comparison of prediction programs with 15 functional variants

Chromosome	Nucleotide position	Reference nucleotide	Variant nucleotide	Gene	rs ID number	Amino acid change^a^	PolyPhen-2	SIFT	MAPP	VarioWatch	Loss of function
2	138476119	C	T	*HNMT*	rs11558538	T105I	D	D	D	D	Yes
4	26092552	C	T	*CCKAR*	rs52795588	V365I	D	T	T	D	Yes
4	165337896	A	G	*ANP32C*	NA	Y140H	D	D	D	D	Yes
5	7923973	A	G	*MTRR*	rs1801394	I49M	D	D	D	D	Yes
7	5993056	C	T	*PMS2*	rs1805324	M622I	T	T	D	T	Yes
7	5993133	T	A	*PMS2*	rs1805318	T597S	T	T	D	T	Yes
7	127041823	G	A	*PAX4*	NA	R121W	D	D	D	D	Yes
7	127042702	G	A	*PAX4*	rs35155575	R37W	D	D	D	D	Yes
10	42930125	G	A	*RET*	rs1799939	G691S	D	T	D	D	No
10	72030393	G	A	*PRF1*	rs35947132	A91V	D	D	D	D	Yes
12	38989178	G	A	*LRRK2*	rs7133914	R1398H	D	T	T	T	Yes
12	39000112	G	C	*LRRK2*	rs33949390	R1628P	D	D	T	D	Yes
12	39043595	G	A	*LRRK2*	rs34778348	G2385R	T	T	D	D	No
13	32911463	A	G	*BRCA2*	**r**s1799944	N991D	T	T	D	D	Yes
19	15851431	C	T	*CYP4F2*	rs2108622	V433M	D	D	T	D	Yes

Correct predictions							11/15	10/15	9/15	10/15	

This table highlights the 15 variants that were a gold standard in a functional study.

^a^ Position of the amino acid change associated with the nucleotide change.

## Discussion

Although the four prediction programs did have some pairwise agreement in identifying deleterious mutations, only 3.5% of variants were classified as deleterious and 10.9% as tolerated across all four programs. The observed differences may be due to different classification cutoff points on the probability of being deleterious but are most likely due to the different methods used to classify variants.

Although functional data are not available for most of the variants examined, they were available for a small number of variants. However, even for variants with functional data, the programs did not provide consistent results. We highlight two examples from Table 3. Kuznetsov et al. [11] used a functional assay to determine that the N991D variant of the *BRCA2* gene was a neutral mutation. A missense variant, N991D was introduced into the cells using Bacterial Artificial Chromosome BACs, and the mutant cells were subjected to various DNA-damaging agents and then compared to control cells. Kuznetsov et al. [11] found that N991D showed no difference in its sensitivity to these agents compared with control cells, whereas cells deficient in the *BRCA2* protein were highly sensitive to these DNA-damaging agents. Thus the N991D mutation was classified as a neutral mutation. However, MAPP and VarioWatch predicted the mutation to be damaging, whereas SIFT and PolyPhen-2 tolerated the mutation. This may reflect the different methods, because MAPP relies primarily on sequence-based features, such as phylogeny and what the amino acid change is in regards to the surrounding sequence. On the other hand, both SIFT and PolyPhen-2 rely heavily on structural features, such as how the variant affects the protein’s tertiary structure and its interactions with ligands. Wilson et al. [12] examined the role of a common mutation at rs1801394 in the gene for methionine synthase reductase (*MTRR*) and its association with an increased risk for spina bifida. Fibroblast lines were isolated from patients with homocystinuria to isolate and identify mutations in the *MTRR* gene using reverse transcription polymerase chain reaction (PCR). Wilson and colleagues found that rs1801394 has the substitution I22M, or I49M, depending on the isomer. A population of individuals with spina bifida and their mothers was analyzed for the presence of this mutation. Two copies of the mutation in mothers resulted in 5 times the risk of their child having a neural tube defect compared to mothers lacking two copies of this mutation. This mutation was predicted to have a deleterious effect in all the programs (Table 3).

Although all four programs had the same relative success in predicting the functional status of the 15 variants for which functional data were available, only about two-thirds of variants were correctly classified. The small sample size of variants would have been more informative if any of the programs had been a distinct outlier, but this was not the case. A more comprehensive look at functional variants using additional exome sequencing data is warranted, as several hundred variants may better discriminate the predictive ability of the programs.

There were some limitations in this study, including a lack of reference sequence. Therefore we used Build 36 from the National Center for Biotechnology Information (NCBI) to determine the location of each variant. However, this assumption resulted in several instances in which the variant provided was identical to the reference allele according to the dbSNP database, showing inconsistencies between the reference sequence for this data set and the substitute reference sequence used from the UCSC Genome Browser. To determine the amino acid changes, we used coordinates of the nucleotide variant that were taken from the GAW17 data, and the nucleotide and amino acid sequences were taken from the CCDS sequence. We also likely missed functional variants, given the search terms in OMIM used to identify functional variants.

## Conclusions

The four programs compared in this analysis (SIFT, MAPP, PolyPhen-2, and VarioWatch) differ greatly in their predictions of the probability that a SNP variant will be functionally deleterious. When selecting variants for experimental or functional follow-up study, a conservative approach, such as SIFT, should be used to minimize false positives. However, if the program is being used to select variants for further statistical analysis, a less conservative approach, such as PolyPhen-2, may be preferred to capture all possible deleterious variants. It is important to note that these programs have different methods and discordant results, so we do not recommend using any program as a true substitute for functional assays.

## Competing interests

The authors declare that there are no competing interests.

## Authors’ contributions

AJ, GW, AC, AG, AM conducted primary analysis of the data. PD and APK conceived of the study and participated in its design and coordination. AJ, GW, PD and APK drafted the manuscript. All authors participated in edited and final approval of the manuscript.
